# Cancer recording in patients with type 2 diabetes in primary care and hospital admission data

**DOI:** 10.23889/ijpds.v1i1.334

**Published:** 2017-04-19

**Authors:** Amitava Banerjee, Jane Burnell, Chris Sutton

**Affiliations:** 1 University College London; 2 University of Central Lancashire

## Objective

The choice of oral anticoagulant(OAC) for stroke prevention in patients with atrial fibrillation(AF) is now between warfarin and four non-vitamin K anticoagulants(NOACs):dabigatran, rivaroxaban, apixaban and edoxaban. Although discontinuation rates were reported in clinical trials of the NOACs, real-world persistence, adherence and cross-over between therapies are unknown.

## Methods

Prescription data for OACs in adults with AF between April 2011 and December 2015 were analysed from a representative national primary care database in England(The Health Improvement Network, THIN). Edoxaban was approved for use in the UK in September 2015 and therefore insufficient numbers of prescriptions were available for analysis. Persistence(estimated by gap between prescriptions) and adherence(proportion of days covered, PDC) for OACs were assessed in individuals with AF newly treated with dabigatran, rivaroxaban, apixaban or warfarin with at least 365 days follow-up after the prescription date. For each NOAC, the proportion of cross-over to another OAC was analysed.

## Results

Among 4,354,740 individuals, 118,056 with AF were identified, of which 82,795 had available prescription data for OACs. Of these patients, 78,447 had at least 12 months data: warfarin (n=67781), dabigatran (n=2540), rivaroxaban (n=5666) and apixaban (n=2460). At 1 year, the crude persistence rates were 88.7% (95% CI 88.4-88.9) for warfarin, 59.1% (57.2-61.0) for dabigatran, 64.7% (63.4-65.9) for rivaroxaban and 67.0% (65.1-68.9) for apixaban. Persistence was significantly lower than in the clinical trials and was lower for NOACs than warfarin.

The adherence rates (PDC>80%) were 87.5% (CI 87.3,87.6) for warfarin, 83.5% (CI 82.3,84.8) for dabigatran, 84.1% (CI 83.1,85.1) for rivaroxaban and 83.6% (CI 81.6,85.5) for apixaban. The proportion of NOAC users crossing over to another NOAC and to warfarin were 12.1% and 7.3% for dabigatran, 3.4% and 3.4% for rivaroxaban, and 1.9% and 1.5% for apixaban respectively.

## Conclusion

In a large English population, NOACs exhibited lower persistence rates than in clinical trials, warranting further analyses of side effect profile and acceptability of OACs. Routinely collected data can be used to measure real-world persistence and adherence which are important in future studies of comparative effectiveness.

